# Not All Levels of Social Re-Inclusion Allow for Recovery from Negative Outcomes of Social Exclusion: The Moderating Role of Self-Esteem

**DOI:** 10.3390/bs14020088

**Published:** 2024-01-25

**Authors:** Beibei Kuang, Sik Hung Ng, Shenli Peng, Ping Hu, Yanqiu Wei

**Affiliations:** 1 College of International Relations, National University of Defense Technology, Nanjing 210039, China; 2 Department of Psychology, Renmin University of China, Beijing 100872, China; ng5566@netvigator.com (S.H.N.); slpeng@hunau.edu.cn (S.P.); weiyanqiu@ruc.edu.cn (Y.W.); 3 College of Education, Hunan Agricultural University, Changsha 410128, China

**Keywords:** social exclusion, social re-inclusion level, self-esteem, recovery effect

## Abstract

Previous studies on social exclusion have focused on its adverse effects, rarely exploring how social re-inclusion can aid recovery from exclusion-induced distress. The level of social re-inclusion that can help individuals recover from social exclusion, and whether the recovery effect is influenced by individual characteristics are unclear. The present experimental study extends the Cyberball paradigm, adding a re-inclusion stage to explore the recovery effects of four levels of social re-inclusion on affect; furthermore, it tests the moderating role of self-esteem in the recovery effect. A total of 154 Chinese college students participated in the experiment. Results showed that (1) recovery was effective when the level of re-inclusion was equal to (replica re-inclusion) or greater than (moderate and high over-re-inclusions) the pre-exclusion level of inclusion, but ineffective when it was below this level (token re-inclusion); (2) the re-inclusion level positively predicted recovery, and this was moderated by self-esteem—the prediction was effective for participants with middle and high self-esteem, but not for participants with low self-esteem. These results are discussed from a group process and self-psychology perspective.

## 1. Introduction

Social exclusion refers to the process of being ostracized, not chosen, or ignored by other individuals or groups [1]. Whatever its form, social exclusion severs human connectedness and adversely affects the cognition [2], behavior [3], and affect [4] of individuals. In recent times, the exclusion in the form of social isolation during the COVID-19 pandemic, widely used as a type of infection control, has damaged the mental health of millions of people worldwide [5,6,7]. Researchers have simulated social exclusion using the Cyberball paradigm [8], which has proven to be effective in demonstrating the harm of even a relatively brief experimentally induced experience of social exclusion [9]. So far, relatively few studies have explored the positive recovery processes from social exclusion, such as social re-inclusion. It is unclear how much social re-inclusion is needed to recover from the negative impact of social exclusion, and whether the recovery effect is identical for all people. The present Cyberball experiment systematically explored the relative recovery impact of four levels of social re-inclusion on affect, and tested the extent to which the recovery effects would be moderated by participants’ self-esteem.

### 1.1. Social Re-Inclusion after Exclusion

In a typical Cyberball experiment, each participant plays a ball-throwing game with co-players who have been instructed or computer-programmed by the experimenter to throw the ball either to themselves and the participant throughout the game (inclusion condition), or only among themselves after several initial throws that included the participant (exclusion condition). In studying re-inclusion, previously excluded participants would participate in a second game, in which one or more co-players would now throw the ball to re-include the participant. So far, re-inclusion experiments have focused only on one level of re-inclusion, which is equal to the level of inclusionary play prior to the exclusion in the first game [10]. A more systematic study of the recovery effect of re-inclusion would have to incorporate other levels for comparison. In principle, the level of re-inclusion may be under or over the level of inclusion prior to exclusion. These two levels can be considered as experimental simulations of tokenism (under-re-inclusion) and positive discrimination or affirmative action (over-re-inclusion) in social debates concerning ageism [11,12], populism [13], sexism [14], as well as employment and educational equalities [15,16]. Thus, their study would have social relevance in addition to providing a more comprehensive framework for exploring the recovery effects of various levels of re-inclusion.

At the core of the re-inclusion concept is the idea of social-psychological repair. Re-inclusion would repair ostracized individuals’ damaged human connectedness with their co-players, leading to an improved perception of the latter and thereby facilitating the process of affect recovery. Higher levels of re-inclusion would have a greater recovery effect because of their greater repair potential. Partial support for this working hypothesis can be inferred from “compensation” research, which compares the recovery effects of different amounts of compensation awarded to victims of damages from financial or service failures. Earlier studies found that equal compensation (i.e., compensation that covered the exact amount of damage) and overcompensation were more effective in repairing trust than partial compensation [17,18]. Further attempts to differentiate between overcompensation and equal compensation found no differences [19]. This negative finding is theoretically interesting in suggesting that overcompensation might have generated uncertainties over its trustworthiness, as indicated by conflicting cognitions and sense-making processes [20]. One implication for the present study is that over-re-inclusion would only work in signifying trustworthiness if it were seen as coming from the unanimous efforts of all co-players, not just from some. Consistent with this implication, the present study manipulated two versions of over-re-inclusion, namely, high over-re-inclusion (abbreviated as HOR henceforth), involving all co-players, and moderate over-re-inclusion (MOR), involving only a majority of co-players. In sum, four levels of re-inclusion were compared in the present experiment: token re-inclusion, equal (or replica) re-inclusion, MOR, and HOR (see Table 1).

### 1.2. Self-Esteem

In psychology, the importance of self-esteem has been recognized since the late nineteenth century [21]. Self-esteem is a person’s evaluation of their worth as a person [22], determined by the ratio of their actualities to their supposed potentialities [23]. Actualities come from success or failure experiences, whereas potentialities point to future expectations. Sociometer theory views self-esteem as a direct indicator of social interaction experiences or interpersonal monitoring [24]. High self-esteem comes from warm or positive social inclusion experiences, whereas low self-esteem often derives from negative social feedback from others. Implicit social cognition theory proposes that past experiences influence judgment in a fashion not introspectively known by individuals [25]. High self-esteem protects against stress, anxiety, and negative social comparisons [26], while low self-esteem is seen as a risk factor [27]. Thus, self-esteem may influence the recovery effect of re-inclusion.

Researchers have explored the moderating role of self-esteem in the relationship between the external environment and outcomes. Self-esteem has been found to facilitate health and positive social behaviors, while buffering the impact of negative influences [28,29] as well as the negative impact of experimentally manipulated threats to body image and appearance [21]. Some studies have focused on the role of self-esteem on the effects of social exclusion, reporting that participants with low self-esteem were more vulnerable to social exclusion than those with high self-esteem [30]. A fMRI study by Keiichi et al. found that low self-esteem enhanced social pain and dorsal anterior cingulate cortex activation in individuals, caused by social exclusion, as opposed to high self-esteem [31]. However, these studies have mainly focused on the impact of self-esteem on social exclusion. The moderating effect of self-esteem on the recovery effect of re-inclusion has yet to be tested. A significant moderating effect can be gleaned from Kong et al., who found that social support positively influenced the life satisfaction and positive affect of participants with high self-esteem, while failing to influence those with low self-esteem [32]. A recent review of several meta-analyses concluded that self-esteem functioned as an adaptive trait with wide-ranging influences on healthy adjustment and adaptation [26]. These results provide a reference point for this study for testing the moderating role of self-esteem on the recovery effect of re-inclusion.

## 2. Materials and Methods

### 2.1. Overview

To explore the effects of the four levels of re-inclusion on post-exclusion affect recovery and test the moderating role of self-esteem in these recovery effects, the following research design was developed by extending the Cyberball paradigm to incorporate a re-inclusion stage following a first stage in which participants played the Cyberball game under an exclusion or inclusion condition. The inclusion condition would serve as a comparison for checking the manipulation of exclusion and testing the effect of exclusion on affect. In the second re-inclusion stage, participants who have experienced social exclusion stayed behind to play a second Cyberball game under one of the four re-inclusion conditions. Their self-esteem was measured as a moderating variable, positive and negative affect were the dependent variables, and the participant’s perception of the other players was used as a manipulation check on re-inclusion. The flow of the experiment was as follows.

- Pre-tests: Prior to the start of the Cyberball game, all participants completed the Positive and Negative Affect Scale (PANAS) and the Self-Esteem Scale.
- Stage 1: Participants played the Cyberball game under either the exclusion or inclusion condition.
- Mid-tests: At the end of Stage 1, all participants repeated the PANAS and completed a Person Perception Questionnaire. Afterwards, participants under the inclusion condition were debriefed and dismissed, and the excluded participants continued to Stage 2.
- Stage 2: Formerly excluded participants played the Cyberball game under either the token, replica, MOR, or HOR condition.
- Post-tests: At the end of the second game, participants completed the PANAS for a third time and the Person Perception Questionnaire for a second time.

### 2.2. Participants

Participants were college students and received course credit for their participation. They all reported normal or corrected-to-normal vision and provided written informed consent on the understanding that they could withdraw from the study at any time. The study was approved by the ethics board of Renmin University of China.

Several participants with incomplete responses to the questionnaires were omitted from the final count of sample sizes, details of which are shown in Table 2 and Table 3. The overall sample consisted of 154 college students (25% male; mean age: 19.66 ± 1.79), out of which 30 were randomly assigned to the inclusion condition, and the remaining 124 to the exclusion condition. The latter continued to Stage 2 and were randomly assigned to the four re-inclusion conditions (token re-inclusion, *n* = 34; replica re-inclusion, *n* = 31; MOR, *n* = 29; and HOR, *n* = 30). A post hoc power analysis showed that the sample size of *n* = 124 has reached the acceptable 80% power for detecting a medium effect size f = 0.25 (equivalent to effect size d = 0.50 or η^2^ = 0.06) at α = 0.05 for the *F* tests that would be used to evaluate the hypotheses [33,34].

### 2.3. Measures

*Self-esteem*. Participants’ self-esteem was assessed using the Chinese version of the Self-Esteem Scale [35], which was revised from Rosenberg’s Self-Esteem Scale [36]. Participants indicated how much they agreed with 10 statements that addressed their general feelings about themselves (e.g., “I feel good about myself”) on a 4-point Likert scale ranging from 1 (*strongly disagree*) to 4 (*strongly agree*); the self-esteem score ranges from 10 to 40. In the present study, the Cronbach’s α for self-esteem scale was 0.81.

*Affect.* Participants’ positive and negative affect were measured using the Chinese version of the PANAS [37], which was originally developed by Watson et al. [38]. Participants rated their immediate affect on 10 positive and 10 negative items (e.g., “interested”, “upset”), scored from 1 (*not at all*) to 5 (*extremely*). Scores of positive and negative affect range from 10 to 50, respectively. In the present study, the Cronbach’s α of positive and negative affect in pre-test was 0.84 and 0.87, respectively. The corresponding figures were 0.90 and 0.88 in the mid-test, and 0.90 and 0.87 in the post-test.

*Perception of other players.* This questionnaire included two subscales: friendliness and hostility. The friendliness scale comprised two items: “How friendly do you feel toward other individuals involved in the game?” and “How warm do you feel toward other individuals in the game?” The hostility scale also comprised two items: “How hostile do you feel toward other individuals in the game?” and “How angry do you feel toward other individuals who are playing the game?” Participants answered each item on a 5-point format ranging from 1 (*not at all*) to 5 (*extremely*). The two items were aggregated for data analysis. A score of 10 indicates maximum friendliness or hostility. In the present study, the Cronbach’s α of the friendliness and hostility scale was 0.86 and 0.78, respectively, in the mid-test, and 0.82 and 0.78, respectively, in the post-test. The two perception subscales would indicate the extent to which re-inclusion has repaired the damage of prior exclusion to human connectedness. As proposed in Section 1.1, only when social-psychological repair has occurred will re-inclusion be able to facilitate emotional recovery. Hence, perceived friendliness and hostility represent a more valid and direct check of the manipulation of re-inclusion compared to simply asking the participants to estimate whether the level of re-inclusion was under, equal to, or over the level of inclusion in the previous stage of the experiment. Furthermore, the scores between the exclusion and inclusion conditions of the subscales served as a supplementary test of the adverse effect of exclusion additional to its effect on affect. Details can be found in Appendix A.

### 2.4. Procedure

Upon arrival at the research laboratory, participants were seated in one of four rooms, given a brief overview of the study, and informed that they would be playing Cyberball online with three other players seated individually in three separate rooms. Each participant could see the other players entering their respective rooms. In fact, each participant would be playing Cyberball with three virtual players whose ball-throwing behaviors were controlled by the Cyberball program [8]. Each Cyberball game comprised 75 throws. Participants were told that “the purpose of this study was to understand mental visualization”, and were instructed “to imagine playing the game in real life”.

*Stage one* (*game 1*). Under the inclusion condition, all virtual players tossed the ball to each other and the participant with equal frequency. As a result, the participant had a 1/3 probability of receiving the ball on each throw. Under the exclusion condition, during the initial 10 throws, the virtual players tossed the ball to the participant and each other in the same manner as in the inclusion condition to give the participant a taste of inclusion. Afterwards, they unanimously excluded the participant and tossed the ball only among themselves. At the end of the game, all participants completed the PANAS again (mid-test) and the Person Perception Questionnaire (mid-test). Those under the inclusion condition were debriefed and dismissed.

*Stage two* (*game 2*). Formerly excluded participants were randomly assigned to the re-inclusion conditions, which were set up by varying the number of supporters (number of virtual players who included the participant in their throws) and the proportion of their throws to the participant. In replica re-inclusion, the number of supporters was 3 and the proportion of re-inclusionary throws was 1/3, the same as in the initial inclusionary play prior to exclusion in the previous game (see Table 1). Under the token re-inclusion condition, 1 supporter directed 2/3 of its throws to the participant. Under the MOR condition, 2 supporters allocated 2/3 of their throws to the participant, while under the HOR condition all 3 supporters did so. At the end of the game, the participants answered the PANAS for the third time (post-test) and the Person Perception Questionnaire for the second time (post-test). Subsequently, they were debriefed and dismissed.

## 3. Results

### 3.1. Stage 1: Being Excluded

*Effect of social exclusion on positive and negative affect.* As the inclusion and exclusion conditions had discrepant sample sizes, their variance homogeneity was checked using Levene’s test ahead of the main analysis. The test confirmed variance homogeneity between the inclusion and exclusion conditions for both positive (*F* (1, 152) = 0.72, *p* = 0.397) and negative (*F* (1, 152) = 2.46, *p* = 0.119) affect. To test the exclusion effect, an analysis of covariance (ANCOVA) was conducted on mid-test affect with the experimental condition (inclusion vs. exclusion) as a between-subjects variable, and with pre-test affect, as a covariate variable. Regarding *negative* affect, there was a significant difference between the inclusion and exclusion conditions (*F* (1, 151) = 6.91, *p* = 0.009, η_p_^2^ = 0.04), with a significant higher mid-test negative affect under the exclusion condition than under the inclusion condition (Table 2). In terms of *positive* affect, no significant difference existed between the two experimental conditions (*F* (1, 151) = 0.17, *p* = 0.679, η_p_^2^ = 0.00). Thus, the adverse emotional impact of social exclusion was specific to negative affect. Supplementary analysis with single items of PANAS as dependent variables can be found in the Supplementary Materials.

*Damage of social exclusion to participants’ perception of other players’ friendliness and hostility.* Levene’s test of variance homogeneity for the exclusion and inclusion conditions found no significant differences for either perceived friendliness (*F* (1, 152) = 0.01, *p* = 0.913) or hostility (*F* (1, 152) = 2.92, *p* = 0.089). The effect of social exclusion on person perception was then tested by independent sample *t*-tests on mid-test perception, with the experimental condition (inclusion vs. exclusion) as an independent variable. Participants under the exclusion condition perceived other players as significantly less friendly (*t* (152) = 3.23, *p* = 0.002, Cohen’s *d* = 0.662) and more hostile (*t* (152) = −3.00, *p* = 0.003, Cohen’s *d* = −0.657; Table 2). These results indicate that exclusion damaged the human connectedness of participants with their co-players, in addition to adversely impacting their affect.

### 3.2. Stage 2: Being Re-Included

*Manipulation check of social re-inclusion: perception of other players’ friendliness and hostility.* To test whether the manipulation of social re-inclusion worked, a two-way mixed repeated-measures analysis of variance (rmANOVA) was performed on perception with test-time (mid- vs. post-test) as a within-subject variable, and re-inclusion (token re-inclusion; replica re-inclusion; MOR; HOR) as a between-subjects variable. The interaction effect was significant for friendliness (*F* (3, 117) = 3.40, *p* = 0.020, η_p_^2^ = 0.08) but not for hostility (*F* (3, 117) = 1.76, *p* = 0.158, η_p_^2^ = 0.04). Planned test-time contrast analysis showed that participants felt significantly more friendly and less hostile in the post-test than in the mid-test for all four social re-inclusion groups (friendliness: all *M_D_* > 1.40, *p* < 0.001; hostility: all *M_D_ >* 1.30, *p* < 0.001), thus confirming the successful re-inclusion manipulation (Table 3).

*Effect of social re-inclusion on improving affect after exclusion.* To test the recovery effect of social re-inclusion, we performed a two-way mixed rmANOVA with test-time (mid- vs. post-test) as a within-subjects variable and re-inclusion level (token re-inclusion, replica re-inclusion, moderate re-inclusion, and high re-inclusion) as a between-subjects variable. Test-time was found to have a significant main effect on *positive* affect (*F* (1, 120) = 8.49, *p* = 0.004, η_p_^2^ = 0.07), with significantly higher post-test than mid-test positive affect (*M_D_* = 1.51, *p* = 0.004). The interaction effect between test-time and re-inclusion level was nonsignificant (*F* (3, 120) = 1.27, *p* = 0.289, η_p_^2^ = 0.03), as was the main effect of re-inclusion level (*F* (3, 120) = 1.94, *p* = 0.127, η_p_^2^ = 0.05). The planned contrast analysis revealed that the post-test positive affect was significantly greater than the mid-test positive affect in the HOR group (*M_D_* = 2.87, *p* = 0.008), but not in the other three groups (all *M_D_* < 1.70, *p* > 0.100) (Table 4). Thus, only when re-inclusion had reached a high over-re-inclusion level supported by all the players it would improve participants’ positive affect.

Regarding *negative* affect, a significant interaction effect between test-time and re-inclusion level was found (*F* (3, 120) = 3.71, *p* = 0.014, η_p_^2^ = 0.09). This indicated that the recovery effects of social re-inclusion differed among the four re-inclusion conditions. A simple effect analysis revealed that post-test negative effect was lower than mid-test negative effect in the replica re-inclusion, MOR, and HOR (*M_D_* = −1.19, *p* = 0.056; *M_D_* = −1.21, *p* = 0.062; *M_D_* = −3.00, *p* < 0.001) groups; however, it was not in the token re-inclusion group (*M_D_* = −0.15, *p* = 0.804; Table 4).

In summary, different levels of social re-inclusion produced different recovery effects. HOR improved both positive and negative affect, replica and MOR improved only negative affect, while token re-inclusion failed completely. Supplementary analysis with single items of PANAS and Perception of other players as dependent variables can be found in the Supplementary Materials.

**Figure 1:**
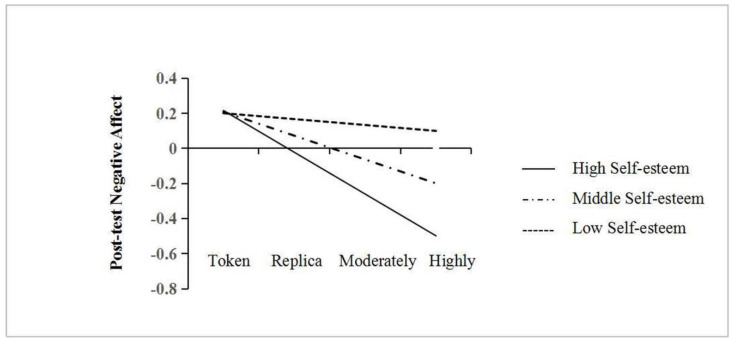
The moderating role of self-esteem in the relation between the social re-inclusion level and post-test negative affect. Note. Token = token re-inclusion, Replica = replica re-inclusion, Moderately = moderate over-re-inclusion (MOR), Highly = high over-re-inclusion (HOR).

*Role of self-esteem in moderating the effect of re-inclusion.* To test the moderating role of self-esteem, the PROCESS macro Model 1 in SPSS was used with 5000 bootstrapped samples [39,40]. Re-inclusion level was set as the independent variable, post-test affect—the dependent variable, self-esteem—the moderator variable, and mid-test affect was taken as a covariate. All variables were standardized. Table 5 reports the results of multiple regression analyses. Regarding positive affect, after controlling for mid-test positive affect, the effect of re-inclusion level was non-significant (*b*_simple_ = −0.30, *t* = −1.91, *p* = 0.059). The results for *negative* affect were more complex. First, after controlling for mid-test negative affect, the re-inclusion level significantly predicted post-test negative affect in the negative direction (*b*_simple_ = −0.14, *t* = −3.19, *p* = 0.002). Second, the interaction effect between the re-inclusion level and self-esteem was significant (*b*_simple_ = −0.10, *t* = −2.21, *p* = 0.029), indicating that self-esteem moderated the relationship between re-inclusion level and post-test negative affect. A moderating analysis was conducted to assess the re-inclusion level’s conditional effect at three values of the moderator (self-esteem): at 1 SD above the sample mean (high self-esteem), at the sample mean (middle self-esteem), and at 1 SD below the sample mean (low self-esteem). The effect of re-inclusion level on post-test negative affect was significant for participants with middle and high self-esteem (*t* = −3.21, *p* = 0.002; *t* = −4.02, *p* < 0.001), but not for participants with low self-esteem (*t* = −0.51, *p* = 0.608). As shown in Figure 1, while the re-inclusion level predicted post-test negative affect in the negative direction for middle- and high-self-esteem participants after controlling for mid-test negative affect, it did not do so for low-self-esteem participants. Notably, the negative direction means that re-inclusion *decreased* post-test negative affect for middle- and high-self-esteem participants. In other words, participants improved their negative affect after experiencing re-inclusion; however, this improvement was significant only for those with middle to high self-esteem.

## 4. Discussion

Previous studies have mainly focused on the recovery effect of replica re-inclusion and have neglected individual differences. The present study explored the recovery effects of four levels of social re-inclusion (token, replica, MOR, and HOR). To our knowledge, it is the first study to simulate a systematic set of re-inclusion levels and to test the moderating role of self-esteem in their recovery effects in a modified Cyberball paradigm.

### 4.1. The Impact of Social Exclusion

This study found that while social exclusion increased participants’ negative affect, it did not decrease positive affect. The results are discrepant from previous studies reporting exclusion-induced damage to both affects [41]; however, they are consistent with others showing a similar over-sensitivity to negative affect [42]. Regarding the non-significant change in positive affect, Fredickson’s broaden-and-build theory suggests a plausible explanation [43]. Positive affect broadens cognitive resources and builds enduring personal resources to cope with difficult life events. Therefore, positive affect tends to be relatively more stable and less subjected to situational fluctuations. This would also explain why only HOR could improve positive affect, whereas the other three levels of re-inclusion could not.

### 4.2. Recovery Effect of Social Re-Inclusion

The most striking finding of the recovery effects of different levels of re-inclusion was that only HOR achieved recovery of both positive and negative affect. To understand this finding, we propose a two-dimensional model of re-inclusion (see Table 6). The includer dimension identifies how many of the three players are acting to include the participant, which may vary from one (minority) to two (majority) or three (unanimity). The second, ball-throw, dimension refers to how often the includer(s) throw the ball to the participant relative to each other. From the perspective of social impact theory [44], the includer dimension corresponds to the number of sources exerting the impact, and the ball-throw dimension corresponds to the strength of their impact. The variations from low to high impact along each dimension can be grouped into three categories. The number of includers may be a minority (low impact), a majority (medium impact), or the entire group (high impact). The entire group engaging in re-inclusionary action is called “consensual group action”. The distribution of ball throws to the participant may be disproportionately low (low impact), equal (medium impact), or large (high impact). High-impact ball throw is referred to as “enthusiastic throw”.

Consensual group action and enthusiastic throw jointly characterize HOR; their combined importance can be viewed in the micro-historical Cyberball context in which re-inclusion occurs. An important feature of this context is the invocation of group-level processes. To recap, re-inclusion (Game 2) was preceded by exclusion (Game 1), during which the other players acted in unison, like a group, in throwing the ball only among themselves. From an intergroup theoretical perspective, the excluded participant might have gradually formed a *group-level* perception or mindset of the game as one that was played by a group of “them” (an outgroup), from whom “me” was being socially disconnected [45,46,47]. The types of re-inclusionary behavior that can mend the damaged human connectedness between the participant and the outgroup would be those that signify that the group, as a whole, was becoming inclusionary. In terms of the two-dimensional model of re-inclusion, such behaviors would involve (a) consensual group action that is (b) enthusiastically (effortfully) enacted, categorized in this study as HOR. Consensual group action and its enthusiastic enactment would be seen as acting in unison to include the participant in the game while demonstrating their genuine intention by making an extra effort to toss the ball more often to the participant than to one another. This level of re-inclusion would be sufficiently powerful to exert its full impact on affect recovery. Consistent with this two-dimensional account, the HOR recovered both positive and negative affect. If re-inclusion lacks either or both consensual group action and enthusiasm, the excluded individuals are less likely to perceive group-level change, and the resulting level of re-inclusion would exert only a partial or even no impact on affect recovery. This is the reason for replica and moderate re-inclusion improving only negative affect, while token re-inclusion recovered neither positive nor negative affect.

### 4.3. The Moderating Role of Self-Esteem

Our study found that the recovery effect of social re-inclusion was moderated by self-esteem. The recovery effect was effective for middle- and high-self-esteem participants, but not for those with low self-esteem. These results are similar to those of Kong et al. in the social support domain [32]. As shown in Table 5, the conditional effects of middle- and high-self-esteem were both significant, the size of which increased from middle (−0.14) to high (−0.24) self-esteem. As moderating effects tend to be small [48], the results from the present exploratory study provide a promising starting point for further research.

Notably, self-esteem might have played its roles in the perception of re-inclusion or in response to participants’ actual experience of re-inclusion. In the former, self-esteem influenced how participants perceived the levels of social re-inclusion, and then impacted their recovery outcomes. In the latter, participants perceived the levels of social re-inclusion as intended, and afterwards, their self-esteem influenced how they reacted to the experience of re-inclusion. Whilst this scenario was supported by results showing the success of the re-inclusion manipulation (Section 3.2), the former scenario cannot be ruled out.

### 4.4. Limitations

Several limitations should be noted when interpreting the results of the current study. First, although any natural change over time was held constant across the four re-inclusion conditions, an additional control condition would have been preferable. For example, we could have randomly assigned some of the post-exclusion participants to a waiting group condition, and afterwards have them re-take the PANAS and person perception questionnaire, comparing these with the re-inclusion results. Second, no qualitative data were obtained to observe participants’ interpretations of the re-inclusion that they have experienced. Post-experimental interviews would shed light on how they view the re-inclusion, and how these interpretations may be linked to their Chinese cultural background. Given that the present study was conducted in mainland China, the results might be different in other cultural contexts. For example, whereas collectivistic Chinese individuals responded positively to replica re-inclusion, individualistic participants reacted resentfully [49]. Furthermore, Chinese participants are less capable of regulating the negative emotions elicited by social exclusion compared to White British [50]. Future cross-cultural research would be necessary to test if these results are replicated in other cultural contexts. Third, the recovery process depicted in the present study may have conveyed an unduly passive view of the human response to social exclusion in real life. Apart from the recovery that is initiated and empowered externally by others, excluded individuals may autonomously seek innovative ways of integration and effective means of persuading or forcing others to become more inclusionary. Recovery strategies that are more autonomous and innovative may bring about more enduring outcomes as well as meaningful social change [16]. Fourth, cross-method validity may be improved by comparing the Cyberball method with alternative methods such as Simulated On-Line Ostracism [51] and cumulative ostracism in daily living [52].

## 5. Conclusions

This study explored the recovery effects of four levels of social re-inclusion (token, replica, MOR, and HOR), being the first (to our knowledge) Cyberball experiment to simulate a systematic set of re-inclusion levels and to test the moderating role of self-esteem in recovery. The results indicated that (a) social exclusion damaged participants’ human connectedness with the other players and adversely affected their affect; (b) re-inclusion repaired the damage and, when its level was greater than or equal to the level of inclusion prior to exclusion (that is, HOR, MOR and replica re-inclusion), reversed the negative effect on affect; and (c) the recovery outcome was moderated by self-esteem.

## Figures and Tables

**Table 1:** Levels of re-inclusion (Stage 2) after being excluded in Stage 1.

Re-Inclusion	Number of Supporters Out of Three	Probability of Receiving Balls from Each Supporting Player	Re-Inclusion Levels (Supporters × Probability)
Under (token) re-inclusion	1	2/3	2/3
Replica re-inclusion	3	1/3	3/3
Moderate over- re-inclusion (MOR)	2	2/3	4/3
High over- re-inclusion (HOR)	3	2/3	6/3

Note. Each participant played the game with three virtual players. The number of supporters was the number of virtual players who tossed the ball to the participant, which varied from 1 (minority) to 2 (majority) or 3 (unanimity). Each virtual player could throw the ball to any one of the other two virtual players or the participant (probability = 1/3). Combining the number of supporters and the proportion of re-inclusionary throws would indicate the overall level of re-inclusion under each condition, as shown in the fourth column.

**Table 2:** Positive and negative affect under each experimental condition, before the start of Game 1 (pre-test) and at its end (mid-test), as well as perceived friendliness and hostility of other players at the end of Game 1.

Experimental Conditions		Positive Affect		Negative Affect		Person Perception	
		Pre-Test	Mid-Test	Pre-Test	Mid-Test	F	H
Inclusion *n* = 30	*M*	30.69	27.27	18.47	15.63	7.23	3.43
	*SD*	5.95	7.25	6.25	5.16	2.13	1.78
Exclusion *n* = 124	*M*	30.23	27.18	19	17.65	5.81	4.77
	*SD*	6.33	7.18	6.56	6.14	2.17	2.27

Note. F = Perceived friendliness of other players; H = perceived hostility of other players.

**Table 3:** Perceived friendliness and hostility of other players before (mid-test) and after (post-test) re-inclusion.

Re-Inclusion		Friendliness		Hostility	
		Mid-Test	Post-Test	Mid-Test	Post-Test
Token (*n* = 33)	*M*	5.35	6.88	5.15	3.67
	*SD*	2.3	1.67	2.56	1.73
Replica (*n* = 31)	*M*	6.13	8.58	4.81	2.71
	*SD*	2.01	1.52	2.21	1.3
Moderately over (MOR) (*n* = 28)	*M*	6.52	8.46	3.86	2.54
	*SD*	2.2	1.14	1.89	0.96
Highly over (HOR) (*n* = 29)	*M*	5.33	8.28	5.17	2.59
	*SD*	2.01	1.69	2.17	0.98

Note. Three participants did not complete the post-test person perception questionnaire. Thus, 121 participants were included in the post-test perception-related analysis, and 124 participants were included in other analyses.

**Table 4:** Positive and negative affect under each re-inclusion condition before (mid-test) and after (post-test) re-inclusion.

Re-Inclusion		Positive Affect		Negative Affect	
		Mid-Test	Post-Test	Mid-Test	Post-Test
Token (*n* = 34)	*M*	26.44	26.5	17.06	16.91
	*SD*	6.2	7.46	5.35	4.83
Replica (*n* = 31)	*M*	27.65	29.26	18.87	17.68
	*SD*	6.74	6.93	6.72	6.1
MOR (*n* = 29)	*M*	29.41	30.93	16.86	15.66
	*SD*	6.93	6.42	4.98	5.6
HOR (*n* = 30)	*M*	25.37	28.23	17.87	14.87
	*SD*	8.52	9.12	7.34	5.88

**Table 5:** Self-esteem moderates the effect of re-inclusion on post-test negative affect: regression model and analysis of conditional effects.

***Predictors***		***b***	***t***
Constant		0.33	2.86 **
Mid-test negative affect		0.79	15.38 ***
Social re-inclusion level		−0.14	−3.19 **
Self-esteem		0.12	1.1
Social re-inclusion level × Self-esteem		−0.10	−2.21 *
*R* ^2^		0.727	
F		77.91 ***	
***Conditional effects***	***effect***		***t***
High self-esteem	−0.24		−4.02 ***
Medium self-esteem	−0.14		−3.21 **
Low self-esteem	−0.03		−0.51

Note. *** *p* < 0.001, ** *p* < 0.01, * *p* < 0.05.

**Table 6:** A two-dimensional typology of re-inclusion in a Cyberball game.

	Includers Distribute Their Ball Tosses		
Number of includers	Disproportionately more to participant (High impact)	Equally (Medium impact)	Disproportionately fewer to participant (Low impact)
Entire group (High impact)	Consensual enthusiastic re-inclusion (**High over-re-inclusion**)	Consensual equal re-inclusion (**Replica re-inclusion**)	Consensual casual re-inclusion
Majority (Medium impact)	Majority enthusiastic re-inclusion (**Moderate over-re-inclusion**)	Majority equal re-inclusion	Majority casual re-inclusion
Minority (Low impact)	Minority enthusiastic re-inclusion (**Token re-inclusion**)	Minority equal re-inclusion	Minority casual re-inclusion

## Data Availability

The data that support the findings of this study are available from the corresponding author upon reasonable request.

## Supplementary Materials

The following supporting information can be downloaded at: https://www.mdpi.com/article/10.3390/bs14020088/s1, File S1: Supplementary analysis.

## Appendix A

### Appendix A.1. PANAS

This scale consists of a number of words that describe different feelings and emotions. Read each item and then mark the appropriate answer in the space next to that word. Indicate to what extent you feel this way right now, that is, at the present moment. Use the following scale to record your answers. 1 = very slightly or not at all; 2 = a little; 3 = moderately; 4 = quite a bit; 5 = extremely.

1. interested
2. distressed
3. excited
4. upset
5. strong
6. guilty
7. scared
8. hostile
9. enthusiastic
10. proud
11. irritable
12. alert
13. ashamed
14. inspired
15. nervous
16. determined
17. attentive
18. jittery
19. active
20. afraid

### Appendix A.2. Person Perception

This scale consists of a number of words that describe person perception. Read each item and then mark the appropriate answer in the space next to that word. Indicate to what extent you feel this way right now, that is, at the present moment. Use the following scale to record your answers. 1 = very slightly or not at all; 2 = a little; 3 = moderately; 4 = quite a bit; 5 = extremely.

1. How friendly do you feel toward other individuals involved in the game?
2. How warm do you feel toward other individuals in the game?
3. How hostile do you feel toward other individuals in the game?
4. How angry do you feel toward other individuals who are playing the game?

### Appendix A.3. Self-Esteem Scale

Below is a list of statements dealing with your general feelings about yourself. Please record the appropriate answer for each item, depending on whether you strongly agree, agree, disagree, or strongly disagree with it. 1 = Strongly agree; 2 = Agree; 3 = Disagree; 4 = Strongly disagree.

1. On the whole, I am satisfied with myself.
2. At times I think I am no good at all.
3. I feel that I have a number of good qualities.
4. I am able to do things as well as most other people.
5. I feel I do not have much to be proud of.
6. I certainly feel useless at times.
7. I feel that I am a person of worth.
8. I wish I could have more respect for myself.
9. All in all, I am inclined to think that I am a failure.
10. I take a positive attitude toward myself.
